# Short Stat5-Interacting Peptide Derived from Phospholipase C-β3 Inhibits Hematopoietic Cell Proliferation and Myeloid Differentiation

**DOI:** 10.1371/journal.pone.0024995

**Published:** 2011-09-20

**Authors:** Hiroki Yasudo, Tomoaki Ando, Wenbin Xiao, Yuko Kawakami, Toshiaki Kawakami

**Affiliations:** Division of Cell Biology, La Jolla Institute for Allergy and Immunology, La Jolla, California, United States of America; University of Michigan School of Medicine, United States of America

## Abstract

Constitutive activation of the transcription factor Stat5 in hematopoietic stem/progenitor cells leads to various hematopoietic malignancies including myeloproliferative neoplasm (MPN). Our recent study found that phospholipase C (PLC)-β3 is a novel tumor suppressor involved in MPN, lymphoma and other tumors. Stat5 activity is negatively regulated by the SH2 domain-containing protein phosphatase SHP-1 in a PLC-β3-dependent manner. PLC-β3 can form the multimolecular SPS complex together with SHP-1 and Stat5. The close physical proximity of SHP-1 and Stat5 brought about by interacting with the C-terminal segment of PLC-β3 (PLC-β3-CT) accelerates SHP-1-mediated dephosphorylation of Stat5. Here we identify the minimal sequences within PLC-β3-CT required for its tumor suppressor function. Two of the three Stat5-binding noncontiguous regions, one of which also binds SHP-1, substantially inhibited *in vitro* proliferation of Ba/F3 cells. Surprisingly, an 11-residue Stat5-binding peptide (residues 988-998) suppressed Stat5 activity in Ba/F3 cells and *in vivo* proliferation and myeloid differentiation of hematopoietic stem/progenitor cells. Therefore, this study further defines PLC-β3-CT as the Stat5- and SHP-1-binding domain by identifying minimal functional sequences of PLC-β3 for its tumor suppressor function and implies their potential utility in the control of hematopoietic malignancies.

## Introduction

The development and homeostasis of hematopoietic cells is controlled by the actions of a multitude of cytokines, growth factors, and hormones [1]. Cell surface receptors bound by these ligands activate several signaling pathways including the Jak-Stat (signal transducer and activator of transcription) pathway. The Jak-Stat pathway plays a crucial role in a number of biological functions by activating transcription of numerous genes [2], [3], [4]. Cytokine stimulation activates Jak kinases through transphosphorylation and results in tyrosine phosphorylation of receptor sites, Stats, and other substrates. Following tyrosine phosphorylation, Stats homo- or hetero-dimerize, translocate to the nucleus, and activate gene expression through sequence-specific response elements. Among seven mammalian Stat proteins (1–4, 5A, 5B, and 6), Stat5 encoded by two closely linked genes, *Stat5a* and *Stat5b*, plays a crucial role in the commitment of embryonic stem cells to hematopoietic cells [5], early hematopoiesis [6], [7], and proliferation and differentiation of myeloid [8] and lymphoid cells [9]. Retroviral transduction of hematopoietic stem cells (HSCs) with constitutively active Stat5 promotes self-renewal, proliferation, and survival of HSCs and induces a lethal myeloproliferative neoplasm (MPN) in mice that have received the transduced cells [10]; constitutively active Stat5 in lymphocytes leads to increased numbers of pro-B and pre-B cells as well as CD4 and CD8 T cells and acute lymphoblastic leukemia [11], [12].

Phospholipase C (PLC) hydrolyzes phosphatidylinositol 4,5-bisphosphate to generate diacylglycerol (DAG) and inositol 1,4,5-trisphosphate (IP_3_). DAG can activate several isoforms of protein kinase C, and IP_3_ can mobilize Ca^2+^. PLC-β isoforms (β1–β4) work as effectors and inactivators for the Gq family of heterotrimeric G proteins. Agonist-stimulated receptors increase exchange of GTP for GDP on Gαq, GTP-bound Gαq engages and activates PLC-β, and PLC-β increases the rate of GTP hydrolysis [13], [14], [15]. These functions of PLC-β determine the duration, amplitude, and on-off cycling of the signaling. Unexpectedly from these G-protein-related studies, we showed that *PLC-β3^−/−^* mice develop a late-onset MPN [16]. The mutant mice have increased numbers of HSCs with increased proliferative, survival, and myeloid-differentiative abilities. These properties are dependent on constitutive activation of the transcription factor Stat5 and can be antagonized by the SH2 domain-containing protein phosphatase SHP-1. PLC-β3 functions as a scaffold to interact with Stat5 and SHP-1 via its noncatalytic C-terminal domain (PLC-β3-CT) to form the multimolecular SPS complex [16]. PLC-β3 facilitates SHP-1-mediated dephosphorylation and inactivation of Stat5. Consistent with this, SHP-1 loss-of-function mutant (*motheaten viable, me^v^*) mice also develop MPN, phenocoping *PLC-β3^−/−^* mice. However, the MPN in *me^v^/me^v^* mice is more rapid and fatal than that in *PLC-β3^−/−^* mice. Moreover, *me^v^/me^v^*, but not *PLC-β3^−/−^*, mice develop anemia, a phenomenon often observed in myelodysplastic syndrome.

The above mouse studies seem relevant to human diseases. Stat5-dependent cooperative transformation by active c-Myc and PLC-β3 deficiency was suggested in mouse lymphomas in *PLC-β3^−/−^* and in *Eμ-myc;PLC-β3^+/−^* mice as well as in human Burkitt's lymphoma cells [16]. The same mechanism for malignant transformation seems to be operative in other human lymphoid and myeloid malignancies.

Similar to other scaffold proteins [17], PLC-β3 consists of modular structures including the pleckstrin homology (PH), four EF hands, split catalytic TIM barrel, C2, and CT domains in this order from the N- to C-termini. PH domains are important for recruiting proteins to different membranes by interacting with phospholipids such as phosphatidylinositol 3,4,5-trisphosphate and phosphatidylinositol 4,5-bisphosphate [18] and proteins such as protein kinase C [19] and the βγ subunits of heterotrimeric G proteins [20], [21], although the PH domains of PLC-b2 or PLC-b3 did not bind several phosphatidylinositols (data not shown); EF hands are Ca^2+^-binding helix-loop-helix domains [22]; C2 domains are involved in calcium-dependent phospholipid binding and in membrane targeting [23], [24]. A recent study identified three Gαq-binding regions in PLC-β3 [25]: the short helix-turn-helix (Hα1 and Hα2) region following the C2 domain and the loop between the TIM barrel and C2 domain are critical for PLC catalytic activity; the third Gαq-interacting region, EF hands 3 and 4, are critical for GTP hydrolysis by Gαq. PLC-β also interacts with and is activated by, the βγ complex. In this study we attempted to identify minimal sequences of PLC-β3-CT required for its tumor suppressor function.

## Results

### Noncontiguous multiple short peptides within PLC-β3-CT bind to Stat5 and SHP-1

**Figure 1:**
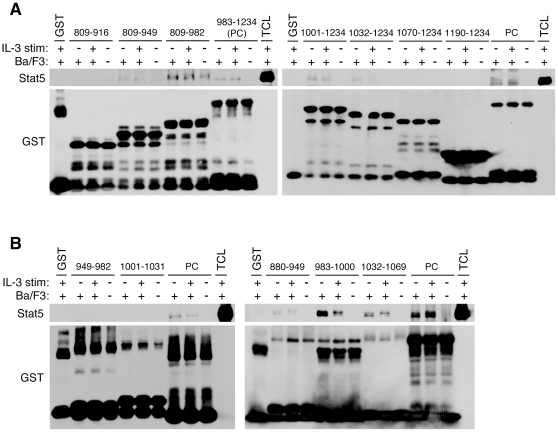
Mapping the Stat5-binding sites within PLC-β3-CT. (A, B) Lysates of Ba/F3 cells incubated with or without IL-3 were mixed with GST fusion proteins, then beads-bound proteins were analyzed by SDS-PAGE followed by immnoblotting with anti-Stat5 antibody. The same blots were reprobed with anti-GST. PLC-β3 portions of GST fusion proteins used are shown by residue numbers. TCL, total cell lysates. The 983–1234 fusion protein (PC) was used as a positive control while GST was used as a negative control.

**Figure 2:**
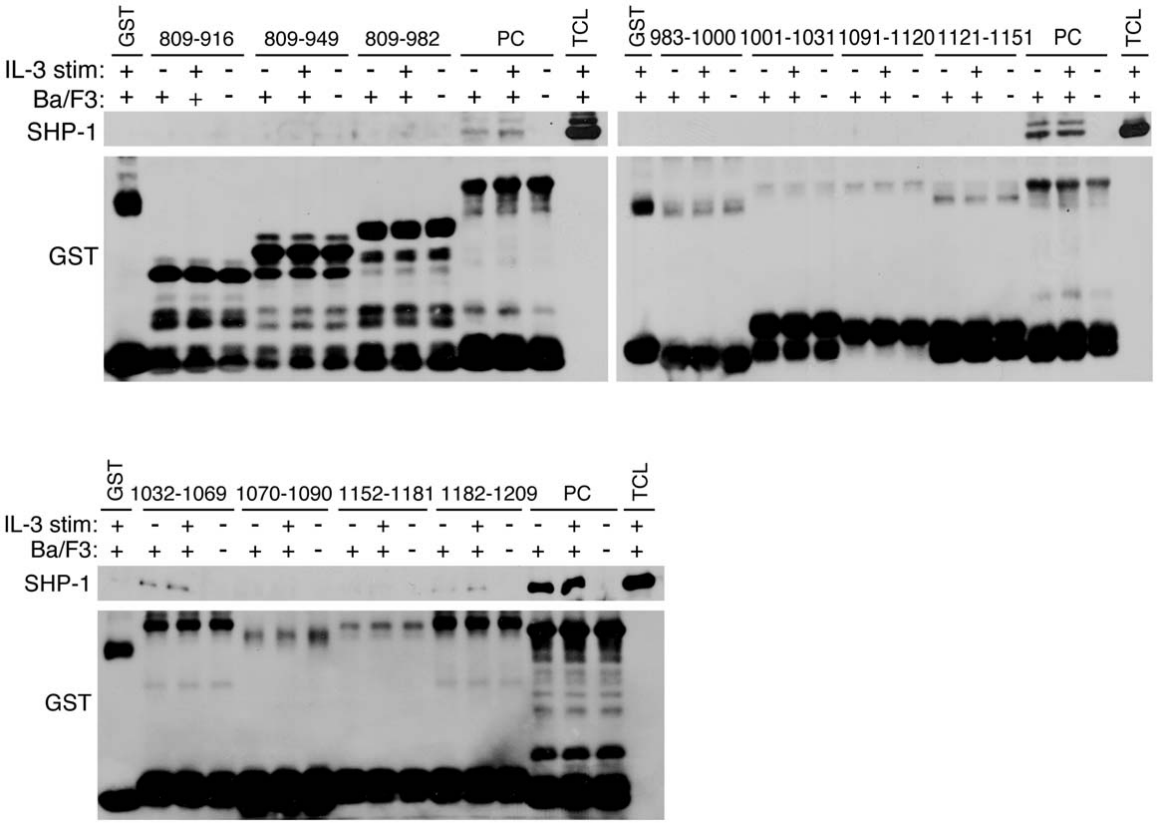
Mapping the SHP-1-binding sites within PLC-β3-CT. GST pulldown experiments were performed similar to those shown in Fig. 1. The 983-1234 fusion protein (PC) was used as a positive control while GST was used as a negative control.

**Figure 3:**
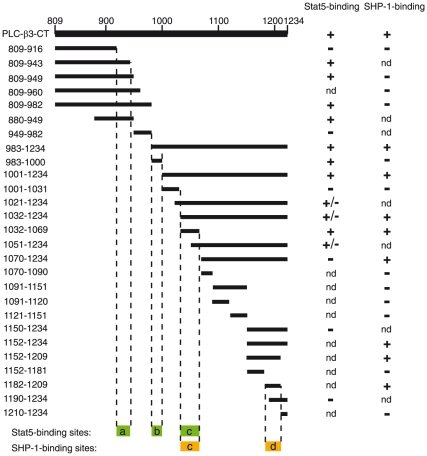
Summary of fine mapping of Stat5- and SHP-1-binding sites within PLC-β3-CT. The deduced Stat5- and SHP-1-binding sites are shown at the bottom. Only GST fusion constructs used to determine the binding sites are shown. nd, not determined.

Our previous study showed that PLC-β3-CT has the tumor suppressor function and contains the interaction sites for Stat5 and SHP-1 [16]. To further our understanding how PLC-β3 performs its growth-suppressive function, we attempted to finely map the Stat5- and SHP-1-binding sites within PLC-β3-CT (residues 809–1234). To this end, we performed pull-down experiments using a large panel of GST fusion proteins containing various portions of PLC-β3-CT. We designed the fusion constructs that allowed us to narrow down the binding sites within less than 40 amino acid residues. Initially, a series of GST constructs with N-terminal deletions and C-terminal deletions were used to map a Stat5-binding site at residues 917-943 (**Fig. 1A** **, Left**) and another at residues 1032–1069 (**Fig. 1A** **, Right**). Using smaller fragments, we could map another Stat5-binding site at 983–1000 (**Fig. 1B**). SHP-1-binding sites were similarly mapped (**Fig. 2**). Results with more than 30 GST fusion proteins are summarized in **Fig. 3**, showing only the constructs essential to determine the binding sites. Three noncontiguous regions, designated a (positions 917–943), b (positions 983–1000), and c (positions 1032–1069), bound to Stat5 and two regions, designated c (positions 1032–1069) and d (positions 1182–1209) bound to SHP-1. Thus, region c could bind both Stat5 and SHP-1.

### Stat5-binding short peptides inhibit the *in vitro* growth of Ba/F3 and TF-1 cells

**Figure 4:**
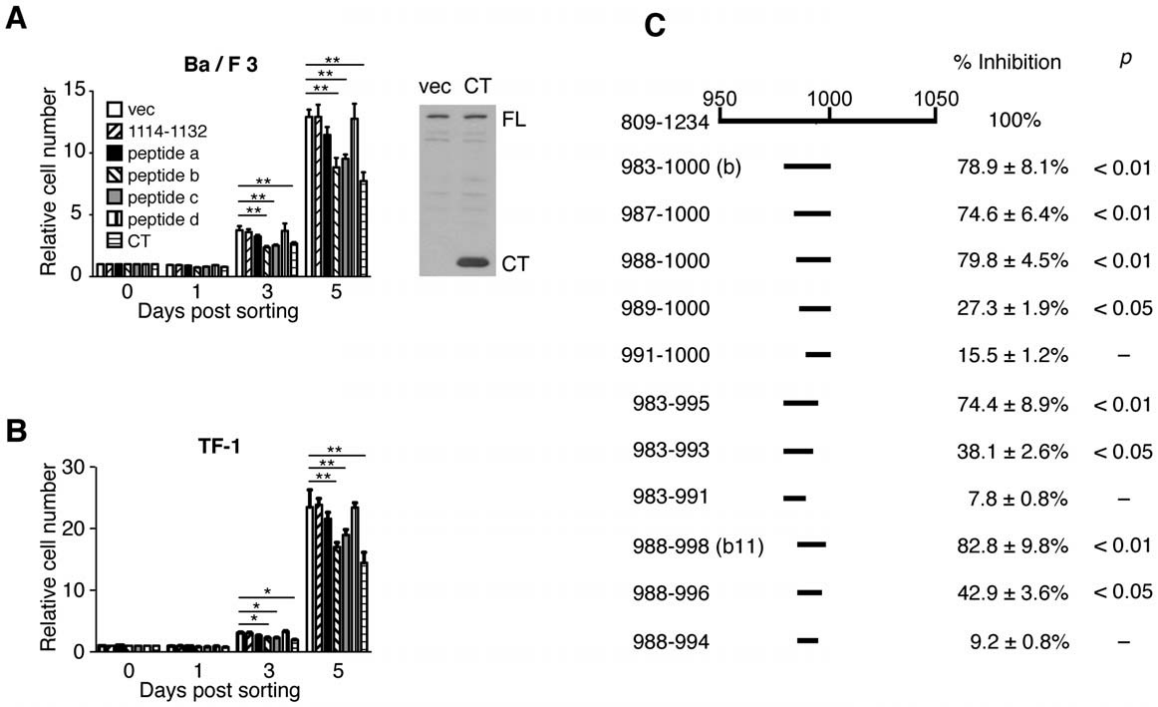
Stat5-binding short peptides inhibit the *in vitro* growth of Ba/F3 and TF-1 cells. Ba/F3 (A, C) or TF-1 (B) cells were retrovirally transduced with the indicated peptides. GFP^+^ transduced cells were FACS-sorted, cultured, and Trypan Blue-resistant live cells enumerated. (C) Growth inhibition of Ba/F3 cells by shorter peptides derived from peptide b. Inhibitory ability was defined as the ratio of (relative cell number in empty vector-transduced cells – relative cell number in peptide-transduced cells) to (relative cell number in empty vector-transduced cells –relative cell number in PLC-β3-CT transduced cells).

Stat5 is a critical transcription factor for the proliferation, survival, and differentiation of HSCs in *PLC-β3^−/−^* mice [16]. Thus, we tested whether overexpression of Stat5-interacting peptides affects the *in vitro* growth of hematopoietic cells. First, these peptides (i.e., peptides a–d) were overexpressed as N-terminally Myc-tagged peptides in Ba/F3 cells. PLC-β3-CT was overexpressed 22 times more than endogenous PLC-β3 in Ba/F3 cells (**Fig. 4A**), as shown previously [16]. Expression of PLC-β3-CT suppressed the IL-3-dependent growth of Ba/F3 cells by ∼40% after 5 days in culture, compared to transduction with empty vector. Importantly, two Stat5-interacting peptides b and c showed significant inhibition of the cell growth (**Fig. 4A**). Similar growth inhibition by peptides b and c was observed with TF-1 erythroleukemia cells (**Fig. 4B**). However, the Stat5-binding peptide a inhibited Ba/F3 cells or TF-1 only slightly. The SHP-1-binding peptide d and a non-Stat5/SHP-1-binding peptide (1114–1132) exhibited no growth-inhibitory activity in Ba/F3 or TF-1 cells. These results suggest that Stat5 binding activity of peptides b and c may be critical for growth suppression. Because peptide b exhibited approximately 80% potency of PLC-β3-CT in Ba/F3 growth inhibition, we tested eleven shorter peptides in this region in the Ba/F3 inhibition assays. Among them, an 11-residue peptide (peptide b11; positions 988–998) exhibited a growth-inhibitory activity as >80% strong as PLC-β3-CT (**Fig. 4C**).

### Expression of the Stat5-interacting peptide b11 inhibits Stat5 phosphorylation

**Figure 5:**
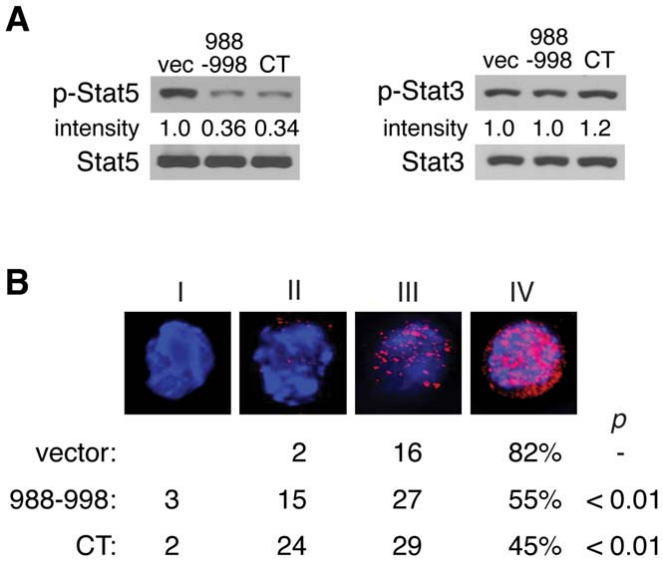
Expression of peptide b11 inhibits Stat5 phosphorylation. Phospho-Stat5 Tyr^694^ levels in Ba/F3 cells transduced with peptide b11 or PLC-β3-CT were measured by immunoblotting (A) and fluorescence microscopy (B) with anti-phospho-Stat5-Tyr^694^. (A) Stat5 amounts were measured by reprobing. Phospho-Stat3-Tyr708 and Stat5 amounts were also measured. (B) Nuclei were stained with DAPI (blue). The patterns of phospho-Stat5 were categorized into four (I-IV) and their distributions are shown by scoring ≥200 cells.

To decipher the molecular mechanism for the peptide b11-induced growth inhibition, IL-3-dependent signaling pathways were analyzed in the tranduced Ba/F3 cells. As shown in **Fig. 5A**, immunoblotting analysis showed that Stat5 phosphorylation at Tyr^694^ was significantly reduced in peptide b11-expressing and PLC-β3-CT-expressing cells. By contrast, Stat3 phosphorylation at Tyr^705^ was not suppressed by peptide b11 or PLC-β3-CT. Immunofluorescence microscopy showed reduced numbers of the peptide b11-expressing and PLC-β3-CT-expressing cells that have high phosphorylation levels of Stat5-Tyr^694^ (**Fig. 5B**).

**Figure 6:**
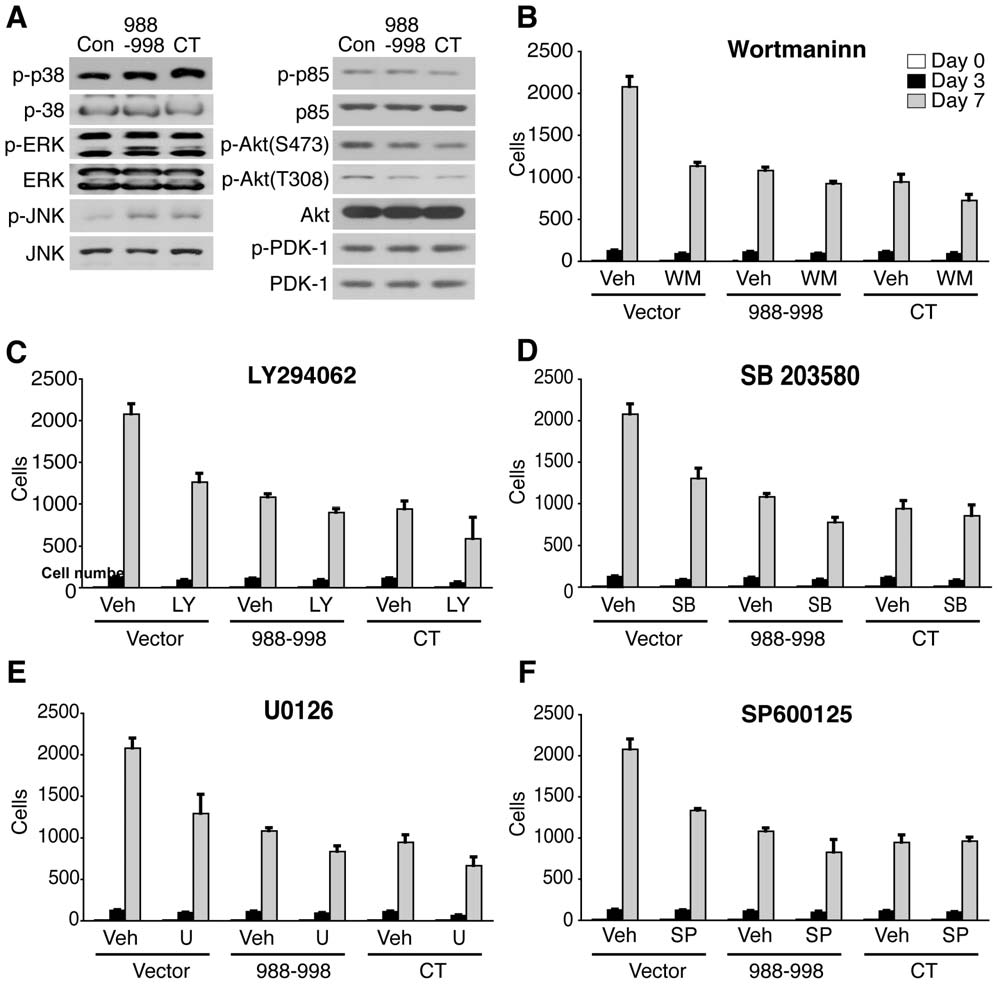
Effects of peptide b11 and PLC-β3-CT on MAPKs and PI3K/Akt pathways. (A) Ba/F3 cells transduced with peptide b11 or PLC-β3-CT were analyzed by SDS-PAGE. Phosphorylation levels of MAPKs and PI3K/Akt were evaluated by immunoblotting. (B) Ba/F3 transduced cells were treated with wortmannin (3 nM), LY294062 (2 µM), SB203580 (0.1 µM), U0126 (1 µM) and SP600125 (0.1 µM). The live cells were counted on day 3 and day 7.

Phosphorylation of Akt (both at Thr^308^ and Ser^473^) was also slightly reduced. By contrast, phosphorylation of MAP kinases (ERK1/2, JNK1/2, p38) at their critical Tyr and Thr residues was rather slightly increased in peptide b11-expressing and PLC-β3-CT-expressing cells, compared to control cells (**Fig. 6A**). Given these results, we evaluated the sensitivity of these cells to inhibitors of the PI3K, ERK, JNK and p38 pathways. The growth of control Ba/F3 cells was inhibited by wortmannin and LY294002 (PI3K inhibitors), U0126 (MEK inhibitor), SP600125 (JNK inhibitor) and SB203580 (p38 inhibitor) in a time-dependent manner (**Fig. 6B**). By contrast, the peptide b11-expressing and PLC-β3-CT-expressing Ba/F3 cells were less sensitive to these inhibitors. These results are consistent with the notion that inhibition of Stat5 is more important than that of other signaling pathways such as PI3K/Akt and MAPK pathways for the suppression of *in vitro* growth of PLC-β3-CT-expressing Ba/F3 cells.

### The Stat5-interacting peptide b11 inhibits myeloid colony formation

**Figure 7:**
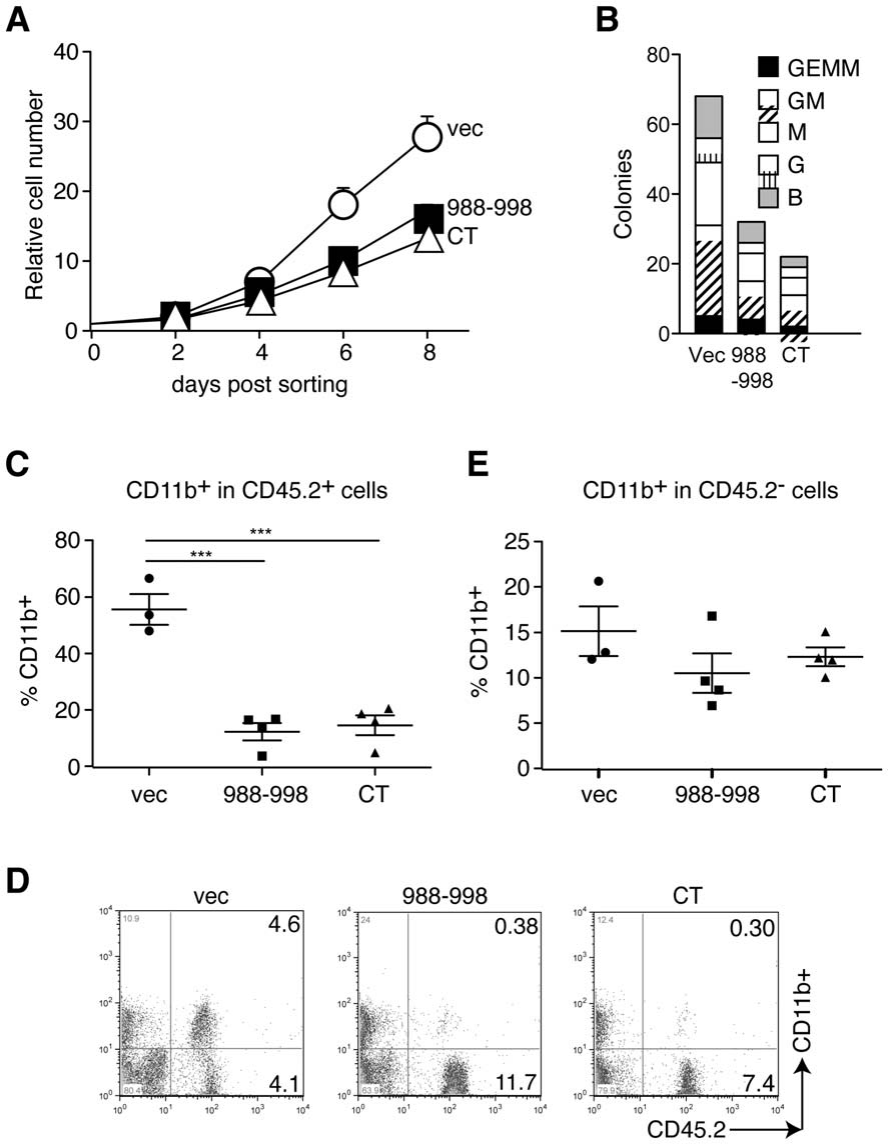
Peptide b11 inhibits *in vitro* and *in vivo* proliferation and differentiation of myeloid cells. (A) Lin^−^ BM cells from old *PLC-β3^−/−^* mice were transduced with retroviruses coding for peptide b11 or PLC-β3-CT, FACS-sorted and cultured in IL-3, SCF and IL-6. (B) The transduced Lin^−^ BM cells were cultured in methylcellulose medium containing SCF, IL-3, IL-6, and EPO. B, G, M, GM and GEMM represent BFU-E, CFU-G, CFU-M, CFU-GM and CFU-GEMM. (C–E) Lin^−^ BM cells from *PLC-β3^−/−^* mice were transduced with the indicated vectors and adoptively transferred to lethally irradiated C57BL/6-Ly5.1 mice. Four months later, peripheral blood was analyzed by flow cytometry (D) for enumeration of CD11b^+^ cells in CD45.2^+^ or CD45.2^−^ cells. ***p<0.001. Numbers in upper and lower right quadrants in panel D represent percentages.

We previously showed that several-fold more myeloid colonies were generated in methylcellulose medium from BM cells or HSCs from *PLC-β3^−/−^* mice than from WT counterparts [16]. Thus, we examined the effect of peptide b11 and PLC-β3-CT on *in vitro* myeloid cell differentiation. Lineage^−^ (Lin^−^) BM cells expressing peptide b11 or PLC-β3-CT grew at reduced rates compared to control cells (**Fig. 7A**) and formed reduced numbers of myeloid colonies (**Fig. 7B**).

### The Stat5-interacting peptide b11 inhibits MPN development

Our previous study demonstrated that retroviral expression of PLC-β3-CT in PLC-β3-deficient CD34^−^c-Kit^+^Sca-1^+^Lin^−^ cells (enriched in long-term HSCs [26]) inhibits the development of MPN when transplanted into lethally irradiated mice [16]. Having shown the strong growth-inhibitory potency of peptide b11, we tested whether expression of this short peptide might suppress the MPN-inducing ability of PLC-β3-deficient HSCs. Lin^−^ BM cells from *PLC-β3^−/^* ^−^ mice (CD45.2^+^) were retrovirally transduced with PLC-β3-CT. When these cells were transferred to lethally irradiated C57BL/6-Ly5.1 mice (CD45.1^+^) together with Sca-1-depleted C57BL/6-Ly5.1 helper BM cells, MPN development was suppressed (**Fig. 7C–D**), as shown previously [16]. >90% CD11b^+^ cells in the peripheral blood were Gr-1^+^ mature granulocytes and 50–60% of CD45.2^+^ cells were GFP^+^. Peptide b11-expressing lin^−^ BM cells also exhibited the ability to suppress MPN development. Thus, peptide b11 transduction also likely targeted long-term HSCs. Interestingly, transplantation of empty vector-, peptide b11- or PLC-β3-CT-transduced cells did not significantly affect the number of host-derived CD11b^+^ cells (**Fig. 7E**).

## Discussion

Using GST fusion pulldown assays, three and two noncontiguous segments of <40 residues were identified as Stat5- and SHP-1-binding sites, respectively, within PLC-β3-CT. When overexpressed, the Stat5-binding peptide b and the Stat5/SHP-1-binding peptide c exhibited an ability to inhibit two hematopoietic cells, i.e., Ba/F3 pro-B cells and TF-1 erythroleukemia cells. Peptide b and its shorter derivative b11 were strong inhibitors with ∼80% potency of PLC-β3-CT. As with PLC-β3-CT, peptide b11 inhibited *in vitro* myeloid cell proliferation and differentiation and suppressed the MPN-inducing ability of *PLC-β3^−/−^* HSC.

Consistent with our previous study [16], reduced Stat5 activity seems to be a molecular basis for the growth-inhibitory function of PLC-β3-CT and its shorter derivatives such as peptide b and b11. The peptides that showed substantial inhibitory activity all had Stat5-binding activity and the cells expressing these peptides showed reduced Stat5-Tyr^694^ phosphorylation. Interestingly, phosphorylation of MAPKs was slightly increased and Akt phosphorylation was slightly decreased in peptide b11- and PLC-β3-CT-overexpressing Ba/F3 cells. These subtle differences detected by immunoblotting might not be detected by flow cytometry of intracellularly stained molecules. Probably, this could be the reason why we failed to observe differences in Akt and MAPKs phosphorylation between WT and *PLC-β3^−/−^* HSCs in our previous study [16]. However, the differences in Akt and MAPKs phosphorylation in peptide b11- and PLC-β3-CT-overexpressing vs. control Ba/F3 cells may not be physiologically relevant. Inhibitor experiments showed that peptide b11- and PLC-β3-CT-overexpressing Ba/F3 cells are less sensitive to growth inhibition by MAPK and PI3K inhibitors than control Ba/F3 cells. These results suggest that IL-3-dependent Ba/F3 cells depend their growth mainly on Stat5, but not MAPKs or Akt. Thus, it will be interesting to examine whether *PLC-β3^−/−^* HSCs are less sensitive to MAPK and PI3K inhibitors than WT HSCs.

**Figure 8:**
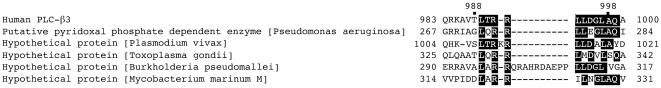
Homology between peptide b of PLC-β3 and hypothetic proteins of parasites and bacteria. BLAST Search was performed using peptide b as a query sequence. Identical residues are shown in a black background.

The C-terminal domain of avian PLC-β is composed of three long helices forming a coiled-coil that dimerizes along its long axis in an antiparallel orientation [27]. All the Stat5-binding and SHP-1-binding sites were mapped to this region, C-terminal to the Gαq-binding helix-turn-helix (Hα1 and Hα2) region. Homology search using the amino acid sequence of peptide b11 (residues 988–998) identified parasite and bacterial sequences with significant homology in their hypothetical proteins (**Fig. 8**), whereas search with the same stringency failed to detect homologous sequences in the genomes of eukaryotes and prokaryotes with peptides a, c, or d (data not shown). If the peptides with homology to peptide b have the ability to inhibit Stat5 activity as shown for peptide b11, such an activity might be important for virulence of the parasites and bacteria.

Introduction of peptides into target cells could be a potential therapeutic strategy for the treatment of cancer. Thus, peptide b11 might have such a potential. However, there seem multiple hurdles for its application. First of all, expression levels of peptide b11 might need to be very high for growth suppression. We used retroviral vector to achieve high-level expression of peptide b11. However, it was difficult to inhibit Ba/F3 cell growth by using a fusion peptide with the TAT protein translocation domain [28](data not shown). Second, targeting specific cells is necessary. These two problems might be resolved by fusing the peptide with a membrane-localizing domain such as PH domain, as an appropriately localized peptide inhibitor may need relatively lower concentrations to be effective. Third, the peptide might need to be stable within target cells.

In summary, this study has identified minimal sequences required for the tumor suppressor function of PLC-β3 that retain the Stat5-binding and Stat5-inhibitory activities. One of the noncontiguous Stat5-binding sequences that was also growth inhibitory was a SHP-1-binding site. However, the second SHP-1-binding site (peptide d) was not growth inhibitory. The growth-inhibitory sequences can be utilized as a potential regulator of hematopoietic cell growth.

## Materials and Methods

### Mice

*PLC-β3^−/−^* mice were described previously [29]. C57BL/6-Ly5.1 mice were purchased from The Jackson Laboratory. This study was carried out in strict accordance with the recommendations in the Guide for the Care and Use of Laboratory Animals of the National Institutes of Health. The Animal Care and Use Committee of the La Jolla Institute for Allergy and Immunology approved all mouse experiments (permit number, AP122-TK1-0410).

### Compounds

Wortmannin, Ly294062, SB203580 and SP600125 were purchased from Calbiochem. U0126 was obtained from Cell Signaling. All drugs were dissolved in DMSO and were diluted to final concentrations of DMSO at less than 0.1%.

### GST Fusion Pull-down Assay

PCR-amplified cDNA fragments using mouse PLC-β3 cDNA as template were cloned into pGEX-3T vector [30]. The fusion protein was induced in DH5α cells by 0.4 mM isopropyl -D-thiogalactoside at 26°C for 3 h and purified by using glutathione-agarose (Sigma). Binding assays were carried out as described [16] with some minor modifications. Ba/F3 cells were lysed with lysis buffer (20 mM Tris-HCl, pH8.0/150 mM NaCl/1 mM EDTA/1% Nonidet P-40) containing protease inhibitors at 4°C for 20 min. Cell lysates from Ba/F3 cells with our without IL-3 stimulation were precleared with GST proteins, then mixed with GST fusion proteins containing various portions of PLC-β3-CT and incubated for 2 h at 4°C with rotation. The binding fraction was washed six times with the lysis buffer without protease inhibitors and loaded on to an SDS/8% PAGE gel. For western blotting, cell lysates were analyzed by SDS-PAGE and followed by immnoblotting with anti-Stat5 (C-17) (Santa Cruz Biotechnology) or anti-SHP-1(C-19) (Santa Cruz Biotechnology).

### Flow Cytometry

Cells were stained with the indicated antibodies. Antibody conjugates used for analysis were obtained from PharMingen (APC-CD11b) or eBioscience (PE-CD45.2.). FACS Calibur (BD Biosciences) was used for analysis.

### *PLC-β3^−/−^* Lin^−^ BM Cell Growth

Lin^−^ BM cells from *PLC-β3^−/−^* mice were sorted using EasySep® Magnet and mouse hematopoietic progenitor cell enrichment kit (EasySep® Stem Cell Technologies) which contained biotin-conjugated CD3, CD11b, CD19, CD45R, Gr-1 and Ter119. *PLC-β3^−/−^* Lin^−^ BM cells were retrovirally transduced with empty-vector, PLC-β3-CT or the Stat5 interactive peptide (988–998). The transduced GFP^+^ cells were sorted into a 96 well plate at a density of 50 cells per well by FACS Diva and cultured in the presence of IL-3, SCF and IL-6.

### Transplantation of Hematopoietic Cells

We transplanted retrovirally transduced Lin- BM cells from old (≥6 months) *PLC-*β*3-/-* mice (CD45.2, 3×10^5^ cells for each recipient) into lethally (960 rad) irradiated C57BL/6-Ly5.1 mice (8-12 week-old) together with 2×10^6^ Sca-1-depleted C57BL/6-Ly5.1 helper BM cells. Sca-1^+^ cells were depleted from BM cells using MACS® and anti-Sca-1 microbeads (Miltenyi Biotech).

### Immunoblotting

Ba/F3 cells transduced with a retrovirus vector (pMIG-PLC-β3-CT or its shorter peptides) were used for the assay. Cell lysates were analyzed by SDS-PAGE and followed by immunoblotting with the following antibodies. Anti-p38 and anti-ERK were purchased from Santa Cruz Biotechnology. Phospho-STAT5 (Tyr^694^) was obtained from Upstate Biotechnology. Anti-JNK, anti-PI3K p85, anti-Akt, anti-Stat3, anti-PDK-1, anti-phospho-p38, anti-phospho-ERK, anti-phospho-JNK, anti-phospho-p85, anti-phospho-Stat3 (Ser^727^), anti-phospho-Akt (Thr^308^), anti-phospho-Akt (Ser^473^) and anti-phospho-PDK-1were obtained from Cell Signaling.

### Retroviral Transduction

Sorted Lin^−^ BM cells were incubated in α-MEM supplemented with 1% FBS, SCF, IL-3 and IL-6 for 24 h, and then transduced with a retrovirus vector (pMIG-PLC-β3-CT or its shorter peptides) using TransIT®-LT1 (Mirus Bio) in the presence of polybrene (7.5 µg/ml) for 24 h. Transduced cells were further subjected to liquid (in the presense of IL-3, IL-6 and SCF) or semi-solid cultures (MethoCult™ M3434 from Stem Cell Technologies). 48 h after transduction, transduced (GFP^+^) Ba/F3 cells and TF-1 cells were sorted by FACS Diva into a 96 well plate at a density of 50 cells per well for enumeration.

### Immunofluorescence

Retrovirally transduced Ba/F3 cells were starved of IL-3 overnight. The next day, the cells were stimulated with IL-3 at 37°C for 5min. The cells were fixed with methanol and incubated for 12 h at 4°C with anti-phospho-Stat5 (clone 8-5-2, Upstate Biotechnology). Then the cells were washed and incubated with Alexa Fluor 568-cojugated goat anti-mouse secondary antibody (Invitrogen) at a dilution of 1∶300 for 1 h at room temperature. Immunofluorescence was observed with a Marianas microscope (Intelligent Imaging Innovations, Inc).

### Colony-Forming Assays

For myeloid colony-forming assays, 2×10^3^ Lin^−^ BM cells or 2×10^4^ Lin^−^ BM cells were transduced with retroviral vectors and GFP^+^ cells were FACS-sorted into MethoCult™ M3434 (StemCell Technologies). After 7–12 days, colonies were scored by microscopy.

### Statistical Analysis

Student's t-test or one way ANOVA were used for statistical analysis. P<0.05 is considered significant.
